# Supplementary material for: Cryo-EM structure of cardiac amyloid fibrils from an immunoglobulin light chain AL amyloidosis patient
Source: Nat Commun. 2019 Mar 20;10:1269. doi: 10.1038/s41467-019-09133-w (PMC6427027; doi:10.1038/s41467-019-09133-w)
Supplement: Supplementary file 1 — Supplementary Information [file 41467_2019_9133_MOESM1_ESM.pdf]

Cryo-EM structure of cardiac amyloid fibrils from an immunoglobulin light chain (AL) amyloidosis patient

P. Swuec, F. Lavatelli et al.

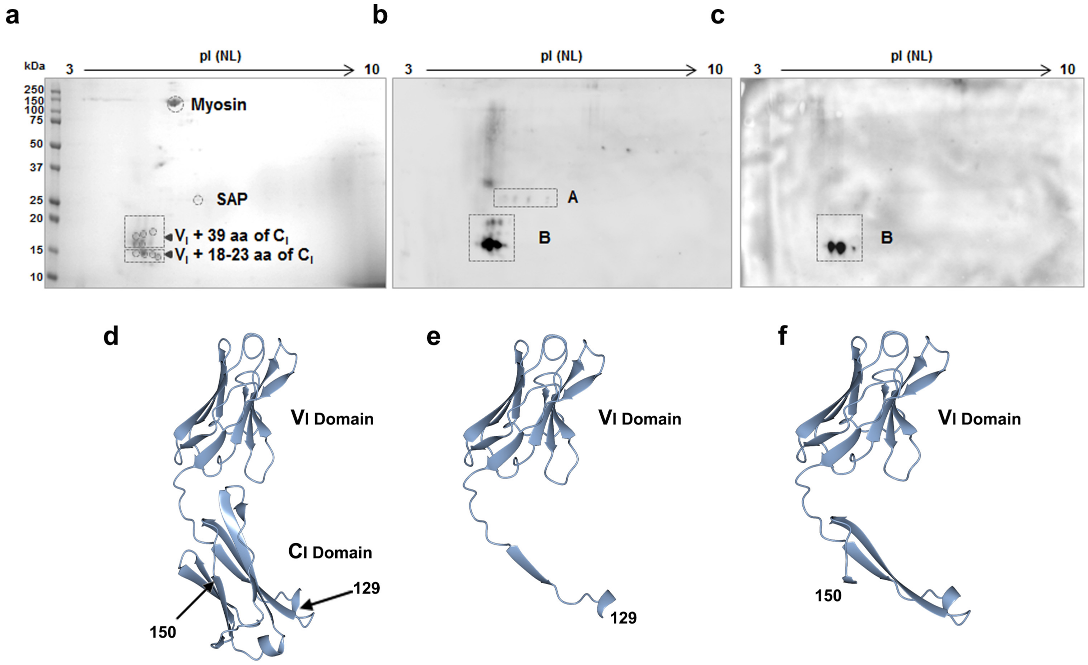

Supplementary Figure 1 | Characterization of AL55 fibrillar deposits. a, Extracted fibrils were solubilised in isoelectrofocusing buffer and characterized by 2D-PAGE: Coomassie staining shows (boxed regions) that fibrils are composed of a complex ensemble of charge isoforms (horizontal trains of spots) and fragments of AL55. This pattern is fully consistent with what previously reported for other AL amyloidosis patients[1]. The nLC-MS/MS identification of the most prominent LC spots (circled) indicates that the most abundant fragments consist of C-terminally-truncated AL55, containing the full V_l and progressively shorter stretches of the constant region (C_l). **b,** the Western blot analysis (polyclonal anti-λ LCs antibody) of the same 2D-PAGE identifies several spots corresponding to the spots identified as C-terminally truncated AL55 fragments (B box). Spots consistent with full length AL55 are also visible (A box).

c, Western blot analysis after limited proteolysis of the extracted fibrils shows that only low MW fragments of AL55 (V_l domain) are resistant to proteolysis, in contrast with high MW fragments and full-length LC. Protein amount loaded in the two Western blots is identical. **d,** Three dimensional model of native full length AL55. **e** and **f,** Three dimensional models of native AL55 where C_l domain is shown up to residues 129 (e) and 150 (f).

Abbreviations: SAP, Serum Amyloid P; pI: isoelectric point; MW: molecular weight; NL, non linear.

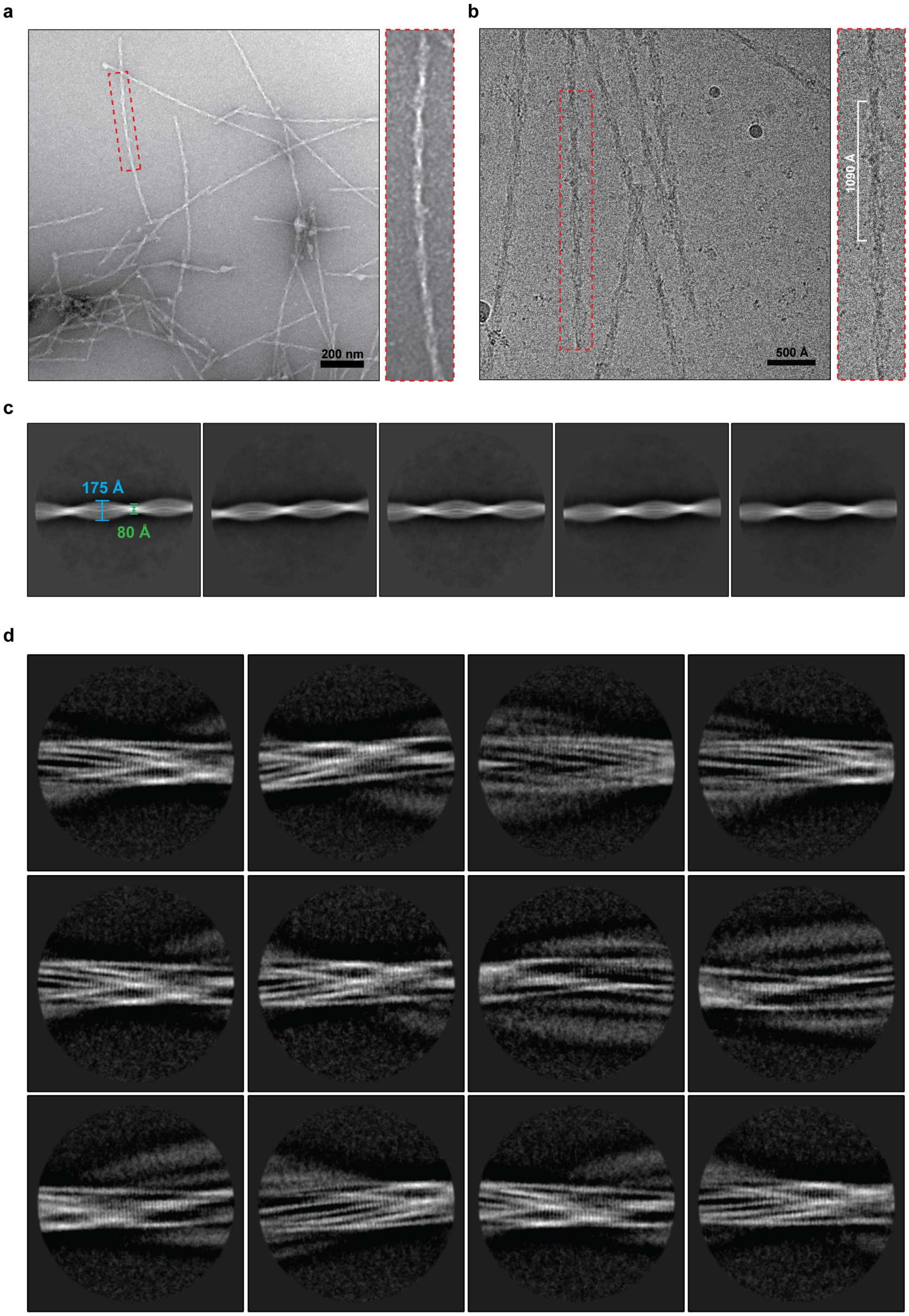

Supplementary Figure 2 | Characterization of AL55 fibrils by electron microscopy. a, Representative micrographs of negative stained and **b,** vitrified AL55 fibrils freshly extracted from hearth in which the helical pitch can be distinguished (dashed red box, right). **c,** The overall topology of vitrified AL55 fibrils was obtained by reference-free 2D classification of segments comprising an entire helical pitch. **d,** The typical cross-β stacking can be

distinguished; averages obtained by reference-free 2D classification using segments comprising 6 asymmetric units and 90% overlap.

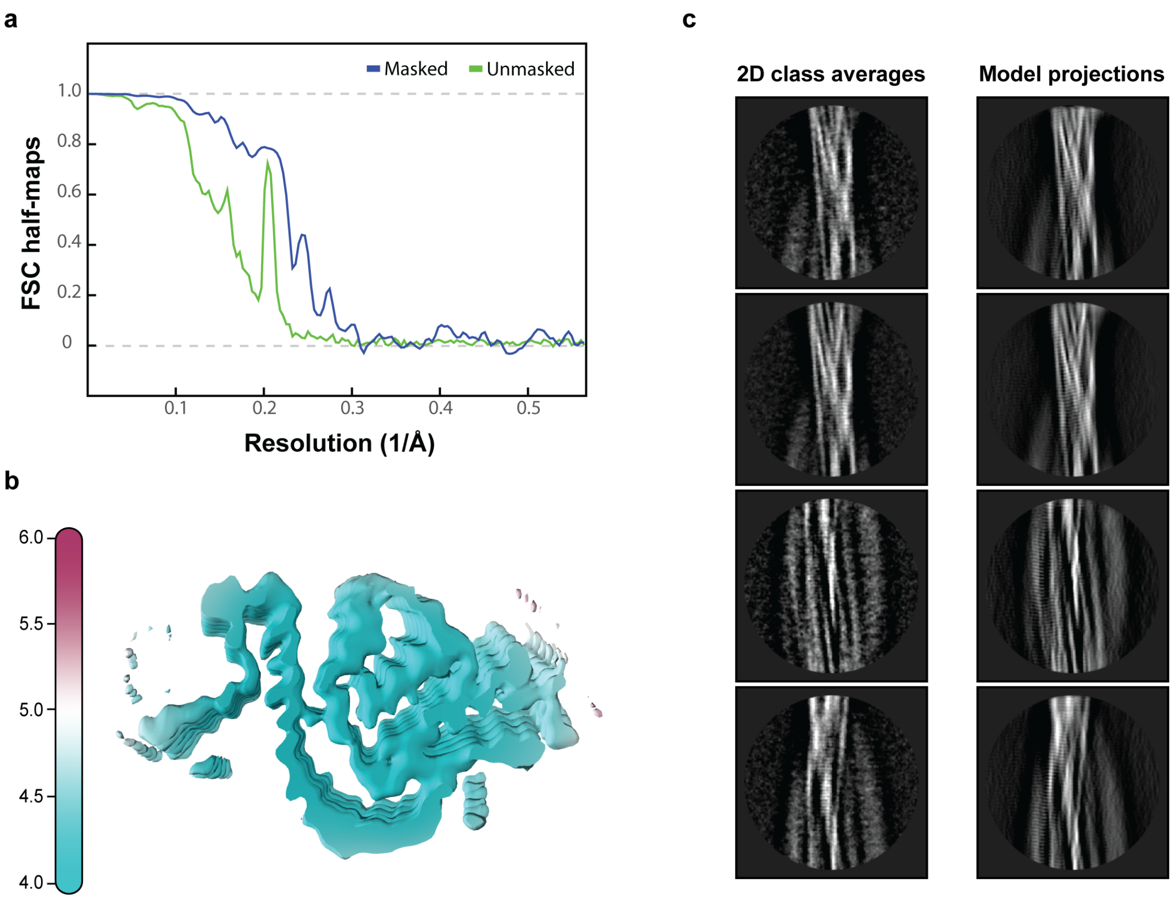

Supplementary Figure 3 | Cryo-EM 3D reconstruction of AL55 fibril. a, Fourier shell correlation curves between the two independently refined half-maps. **b,** Local resolution estimation for AL55 3D reconstruction. **c,** Comparison of 2D projections of the final unsharpened AL55 map with reference-free 2D class averages.

Supplementary Table 1 | Baseline clinical features of AL55 patient

Clinical features	
**Sex, age at disease onset**	M, 60
**Diagnosis**	AL λ amyloidosis
**Organs clinically involved by amyloid disease***	Heart, kidney
**Monoclonal component**	IgGλ + FLCλ
**Serum λ FLC/dFLC (mg/l)**	73.4/55.7
**κ/λ FLC ratio**	0.24
**NT-proBNP (ng/l)**	3,941
**cTnI (ng/ml)**	0.15
**IVS/PW (mm)/EF (%)**	19/19/45%
**Cardiac stage°**	III
**Serum creatinine (mg/dl)**	1.23
**eGFR (ml/min x1.73 m^2)°°**	63
**Proteinuria (g/24h)**	15.9
**Renal stage°°°**	II
Biochemical features of the amyloidogenic monoclonal λ light chain	
**Germline gene**	IGLV6-57
**Calculated MW (Da)**	23306.63
**Calculated *pI***	5.5
**Number of aa**	217

* according to Gertz MA et al, Am J Hematol. 2005;79(4):319-28.
° According to Dispenzieri et al, J Clin Oncol. 2004;15;22(18):3751-7.
°° According to CKD-EPI equation
°°° According to Palladini et al, Blood. 2014;124(15):2325-32.

Abbreviations and symbols: M, Male; FLC, Free Light Chains (quantified using Freelite assay, The Binding Site, Birmingham, UK); NT-proBNP, N-terminal fragment of B-type Natriuretic Peptide; cTnI, cardiac Troponin I; IVS, Interventricular Septum; PW, Posterior Wall; EF, Ejection Fraction; eGFR: estimated Glomerular Filtration Rate; MW: Molecular Weight; pI: Isoelectric Point.
Reference ranges: serum λ FLC <26.3 mg/l, κ/λ FLC ratio 0.26-1.65; serum creatinine <1.18 mg/dl; NT-proBNP <227 in men >50 years, TnI <0.04 ng/ml.

Supplementary Table 2 | Cryo-EM data collection, structure determination and refinement statistics.

**Data Collection**	
Microscope	Talos Arctica
Voltage (kV)	200
Camera	Falcon 3EC
Magnification	×120,000
Pixel size (Å)	0.889
Defocus range (μm)	-0.5 to -2.5
Total electron dose (e^-/A^2)	50
Exposure time (s)	1
Number of frames	39
**Reconstruction**	
Micrographs	1680
Box-size (pixels)	320
Inter-box distance (Å)	32
Segments (total)	104,689
Segments (final reconstruction)	21,031
Resolution (Å)	4.0
Map sharpening β-factor	-106
Helical rise (Å)	4.903
Helical twist (^{o})	-1.608
Symmetry	C1
**Model Composition and Statistics**	
Non-hydrogen atoms	2905
Subunits	5
CC mask	0.747
RMSD bonds (Å)	0.008
RMSD angles (˚)	1.24
All-atom clash score	5.62
Ramachandran outliers (%)	0
Ramachandran favoured (%)	91.8
Rotamer outliers	0
C-beta deviations	0
EMRinger score	5.14
EMRinger Zscore	8.21
Molprobity score	1.80

Supplementary References

1 Lavatelli, F. *et al.* Amyloidogenic and associated proteins in systemic amyloidosis proteome of adipose tissue. *Molecular & cellular proteomics : MCP* **7**, 1570-1583, doi:10.1074/mcp.M700545-MCP200 (2008).
